# Caregiver Responses to Child Posttraumatic Distress: A Qualitative Study in a High‐Risk Context in South Africa

**DOI:** 10.1002/jts.22215

**Published:** 2017-10-27

**Authors:** Victoria Williamson, Ian Butler, Mark Tomlinson, Sarah Skeen, Hope Christie, Jackie Stewart, Sarah L Halligan

**Affiliations:** ^1^ Department of Psychology University of Bath Bath United Kingdom; ^2^ Department of Humanities and Social Sciences University of Bath Bath United Kingdom; ^3^ Department of Psychology Stellenbosch University Stellenbosch South Africa; ^4^ Psychiatry and Mental Health University of Cape Town Cape Town South Africa

## Abstract

Many low‐ and middle‐income countries (LMIC) have high rates of child trauma exposure and limited access to psychological services. Caregivers are often a child's key source of support following trauma in such contexts. The aim of this study was to explore the experiences of primary caregivers in supporting their child posttrauma. Qualitative interviews were conducted with 20 female caregivers from a high‐risk settlement in Cape Town following child trauma exposure. Children were exposed to significant traumatic events, including gang violence, assault, and fatalities of close relatives. The data were analyzed using thematic analysis; several key themes emerged. First, caregivers were typically aware of child distress posttrauma, based primarily on manifest behaviors. Second, caregivers identified varied ways of providing support, including being warm and responsive; seeking to ensure physical safety by encouraging the child's perceptions of the community as dangerous; and encouraging forgetting as a way of coping, with limited discussions of the event. Third, many barriers existed to accessing psychological treatment, and caregivers had low involvement in any interventions. Finally, caregivers also experienced significant distress that could impact their responses to their child. The results illustrate the challenges faced by caregivers in supporting children following trauma in LMIC contexts and the need for accessible psychological interventions.

## Resumen

Respuesta de los Cuidadores al Sufrimiento Postraumático Infantil: un Estudio Cualitativo en un Contexto de Alto Riesgo en Sudáfrica

RESPUESTA DE LOS CUIDADORES LUEGO DEL TRAUMA INFANTIL

Muchos países de bajos y medianos ingresos (LMIC) tienen altas tasas de exposición infantil al trauma y acceso limitado a servicios psicológicos. Los cuidadores son generalmente una fuente clave de soporte para los niños luego del trauma en dichos contextos. El objetivo de este estudio fue explorar las experiencias de los cuidadores primarios al ofrecer soporte a sus niños, después del trauma. Se realizaron entrevistas cualitativas a 20 mujeres cuidadoras de un asentamiento de alto riesgo en Ciudad del Cabo, luego de la exposición al trauma de sus niños. Los niños estuvieron expuestos a eventos traumáticos significativos, que incluyeron violencia de pandillas, asalto y muerte de parientes cercanos. Los datos fueron analizados usando análisis temático; y varios temas clave emergieron. Primero, las cuidadoras estaban típicamente al tanto del sufrimiento post‐trauma del niño, basado principalmente en comportamientos manifiestos. Segundo, las cuidadoras identificaron variadas formas de proveer soporte, que incluían: ser cálidas y receptivas, garantizar seguridad física a través del fomento de las percepciones del niño de la comunidad como peligrosa; y fomentar el olvido como una forma de afrontamiento, con limitadas discusiones del evento. Tercero, existían muchas barreras para el acceso a tratamiento psicológico, y las cuidadoras tenían baja participación en cualquier intervención. Finalmente, las cuidadoras también experimentaron sufrimiento significativo que podría impactar las respuestas a sus niños. Los resultados ilustran los desafíos que enfrentan los cuidadores al proveer soporte a sus niños luego de un trauma en contexto de LMIC y la necesidad de intervenciones psicológicas accesibles.

## 抽象

Traditional Chinese

標題: 保姆對兒童創傷後悲痛的反應:在南非一個高風險環境進行的非量化研究

撮要: 很多低至中收入國家(LMIC)有高水平的兒童受創率, 但缺乏心理治療服務。保姆經常是兒童受創後取得支持的主要來源。本研究探查在兒童受創後, 主要的保姆提供支持的經歷。我們在開普敦一個高風險的聚居地, 對20名女性保姆進行有關兒童受創經歷的非量化面談。那些兒童經歷了顯著的創傷事件, 包括幫派暴力、襲擊和近親死亡。我們就數據採用主題分析, 找出數個關鍵主題。首先, 保姆一般留意到兒童的創傷後悲痛, 主要是基於兒童展現的行為。第二, 保姆會透過數種方法為兒童提供支持, 包括:表現親切, 積極地教導兒童認知該社群的危險性, 以確保兒童的人身安全;鼓勵兒童以遺忘來應對, 減少提及事件。第三, 有很多障礙存在令兒童難以尋求心理治療, 而保姆在任何干預裡的參與度都很低。最後, 保姆亦會感到顯著的悲痛, 那可能影響她們給予兒童的反應。結果反映了LMIC的保姆為受創兒童提供支持所面對的困難, 以及這種環境對心理治療的需要。

Simplified Chinese

标题: 保姆对儿童创伤后悲痛的反应:在南非一个高风险环境进行的非量化研究

撮要: 很多低至中收入国家(LMIC)有高水平的儿童受创率, 但缺乏心理治疗服务。保姆经常是儿童受创后取得支持的主要来源。本研究探查在儿童受创后, 主要的保姆提供支持的经历。我们在开普敦一个高风险的聚居地, 对20名女性保姆进行有关儿童受创经历的非量化面谈。那些儿童经历了显著的创伤事件, 包括帮派暴力、袭击和近亲死亡。我们就数据采用主题分析, 找出数个关键主题。首先, 保姆一般留意到儿童的创伤后悲痛, 主要是基于儿童展现的行为。第二, 保姆会透过数种方法为儿童提供支持, 包括:表现亲切, 积极地教导儿童认知该社群的危险性, 以确保儿童的人身安全;鼓励儿童以遗忘来应对, 减少提及事件。第三, 有很多障碍存在令儿童难以寻求心理治疗, 而保姆在任何干预里的参与度都很低。最后, 保姆亦会感到显著的悲痛, 那可能影响她们给予儿童的反应。结果反映了LMIC的保姆为受创儿童提供支持所面对的困难, 以及这种环境对心理治疗的需要。

Posttraumatic stress disorder (PTSD) can result in negative long‐term outcomes in children (Moroz, 2005). In many low‐ and middle‐income countries (LMIC), defined as countries with a gross national income per capita of USD 12,475 or less (World Bank Independent Evaluation Group, 2007), young people are vulnerable to trauma exposure as a result of several factors, including internal displacement, poverty, and political violence (Hofman, Primack, Keusch, & Hrynkow, 2005; Masinda & Muhesi, 2004). In such contexts, formal psychological services are often extremely limited (Saxena, Thornicroft, Knapp, & Whiteford, 2007). Consequently, a child's family is likely to be his or her key source of support following trauma (Tol, Song, & Jordans, 2013). Previous research has found posttrauma parenting behaviors to be associated with child PTSD. For example, parental warmth and support are thought to facilitate child coping posttrauma by modeling adaptive coping responses and providing a sense of security (Marsac, Donlon, Winston, & Kassam‐Adams, 2013). Conversely, negative parenting behaviors, including overprotection, may increase a child's perceptions of vulnerability to threat, and are associated with higher levels of child PTSD symptoms (Chorpita & Barlow, 1998; Williamson et al., 2017). Parental advocacy of avoidance has also been found to be associated with poorer child outcomes posttrauma (Ehlers, Mayou, & Bryant, 2003). However, the majority of research examining the association between parenting and child PTSD has been conducted in comparatively low‐risk Western samples. Consequently, little is known about the experiences of caregivers in supporting their children in higher risk LMIC contexts, where exposure to violence and other adversities is the norm.

One such LMIC context is the township of Khayelitsha in Cape Town, South Africa. Khayelitsha is home to around one million residents, the majority of whom live in makeshift housing, with an unemployment rate of approximately 51% (Brunn & Wilson, 2013). This periurban settlement has extremely high rates of violent crime, and more than 80% of community youth report exposure to severe trauma (Shields, Nadasen, & Pierce, 2008). The prevalence of PTSD in youth from such communities is correspondingly high, approximately 20%–38% (Das‐Munshi et al., 2016; Seedat, Nyamai, Njenga, Vythilingum, & Stein, 2004), and PTSD is one of the most prevalent diagnoses presenting to local psychiatric services (Traut et al., 2002). Despite the high rates of child trauma exposure, there is a substantial gap between the number of individuals who require mental health treatment and those who receive it in South Africa (Williams et al., 2008), and few medical professionals receive training in appropriate care for victims seeking help following trauma (Gevers & Abrahams, 2014). Families are likely to provide the majority of support posttrauma. As such, the objective of our study was to gain an understanding of caregiver responses to child trauma in high‐risk environments where standard support mechanisms may not be feasible. We specifically aimed to explore: (a) caregivers’ understanding of their child's posttrauma distress; (b) caregivers’ experiences of providing support to their child following trauma exposure; and (c) caregivers’ perceptions of formal support and any barriers to accessing such support.

## Method

### Participants

We conducted a qualitative study of 20 families living in the township of Khayelitsha in Cape Town, South Africa. We recruited primary caregivers of children aged 6 to16 years who had experienced a potentially traumatic event in the past two years (see Table 1). *Primary caregiver* was defined as the individual who serves as a parental figure to the child and is responsible for the child's daily care. Exclusion criteria included existing organic brain damage or intellectual disability in the child precluding mainstream schooling; caregiver being unaware of the child's trauma; child registered with child protection; concerns that the respondent caregiver was the perpetrator of the trauma; and the child being orphaned by the traumatic event.

**Table 1:** Sample Characteristics

Characteristic	*M*	*SD*	*n*	%
Child age (years)	11.50	3.02		
Caregiver age (years)	41.25	8.02		
Caregiver relationship to the child				
Mother			17	85.0
Grandmother			1	5.0
Aunt			2	10.0
Caregiver marital status				
Single			8	40.0
Married/living with partner			8	40.0
Divorced/separated/widowed			4	20.0
Days in the last week child has gone hungry^a^	1	0–5		
Months since trauma	13.9	8.1		
Total traumatic events child experienced^a^^,^ b	3	1–7		
Income^c^				
ZAR 0–2000			8	40.0
ZAR 2001–5000			8	40.0
More than ZAR 5000			3	15.0
Not reported			1	5.0

Note. ^a^Values in the *M* column are presented as median, and values in the *SD column* are presented as a range. ^b^Median number of traumatic events experienced was calculated from caregiver‐report using Part 1 of the UCLA‐Reaction Index (Pynoos et al., 1998). ^c^The minimum living wage in South Africa is industry‐specific; for example, the Ministry of Labour set the minimum living wage for farm laborers at ZAR 2274 per month (138 USD, based on ZAR–USD exchange rate as of 23rd January 2016).

We used opportunity sampling, with community members, church leaders, and nongovernmental organizations being informed about the study and asked to distribute study details to known families who met the inclusion criteria. Families then contacted the research team for more information about the study. The purposive sampling method of snowballing (Heckathorn, 2011) was also used, because participating caregivers often referred researchers to other families with a child or children who had experienced trauma. Researchers met with participants to confirm their eligibility in line with study inclusion and exclusion criteria, provided potential participants with information about the study, and obtained informed consent from caregivers who were willing to take part.

Participating caregivers provided informed consent before taking part. Information sheets/consent forms were translated into Xhosa and back‐translated into English to ensure accuracy (Brislin, 1970), and were read aloud to participants. Prior to signing the consent form, participants were asked to summarize the study in their own words and were invited to ask questions. Any misunderstandings relating to the study procedures, potential risks, or benefits were addressed. All participants were provided with a copy of the consent form and researcher contact details, and were informed that they could withdraw at any time.

A total of 25 caregivers were approached to take part in the study, 20 of whom were recruited. Caregivers who did not participate were either not contactable or did not have time to participate.

### Procedure

Assessments, described below, were conducted face to face by female data collectors in Xhosa, the primary local language at a research centre in Khayelitsha. Several steps were taken to ensure confidentiality, such as storing data by numeric code versus personal identifier. Data collectors first administered questionnaire assessments and then conducted the qualitative interview. Questionnaire items were administered verbally and data collectors recorded responses by hand. Data collectors received a week of training in qualitative interview methods, interviewing trauma‐exposed individuals, and risk and referral procedures. Training was provided by an experienced qualitative researcher with a background in trauma research (JS), and included sessions on the nature, purpose, and conduct of qualitative research, the possible impact of trauma on individuals, in‐depth discussions regarding the aim of each interview question, and mock interviews and feedback from the research team. Detailed feedback on interview content was given through weekly supervision, and quality checks of interviews were conducted throughout data collection.

Caregivers were given a ZAR 120 (approximately USD 8.23) voucher for their participation in the study, the standard amount required by Stellenbosch University Health Research Ethics Committee. Following the interview, all caregivers were given the opportunity to discuss their experience and ask any questions, and offered a letter of referral to local mental health services for themselves or their child (if they desired to pursue such services). A standard protocol for managing risks was in place, approved by the ethics committee, but no risk events were experienced. The study received approval from the University of Bath Ethics Committee (Bath, United Kingdom) and the Stellenbosch University Health Research Ethics Committee (Stellenbosch, South Africa).

### Measures

#### Trauma History

Caregivers were administered Part 1 of the UCLA PTSD Reaction Index (UCLA‐RI; Pynoos, Rodriguez, Steinberg, Stuber, & Frederick, 1998) to assess the child's exposure to traumatic events in the past two years. Part 1 is comprised of a list of 13 possible child exposures (including community violence, natural disaster, abuse, and medical trauma) to which the caregiver responds yes (*present*) or no (*absent*). The resultant score is a count of events. The UCLA‐RI has been widely used internationally (e.g. Murray et al., 2011).

#### Semistructured Interview

The content of the semistructured interview guide was informed by the literature on parent and child experiences posttrauma (DiCicco‐Bloom & Crabtree, 2006; Gill, Stewart, Treasure, & Chadwick, 2008), and by two focus groups conducted with members of the local community to ensure all questions were culturally relevant and sensitive. The interview guide was comprised of demographic items followed by open‐ended questions relating to a caregiver's perceptions of his or her child's posttraumatic distress; the caregiver's experiences of supporting the child posttrauma; the impact of the child's trauma on the family; the caregivers’ perceptions of support; and any barriers to the provision of support. Interview questions included: “Were there any changes you noticed in your child after the event?”; “Do you feel able to support your child after the trauma?”; and “What have other families in this community done to support their children who have had similar experiences?” Interviews were audiorecorded and transcribed verbatim. Transcripts underwent a three‐part transcription and translation process to ensure accuracy, trustworthiness, and credibility (Esposito, 2001). First, the audiorecording was translated and transcribed by an independent, bilingual transcriber who did not conduct the interview. Second, the data collector who conducted the interview reviewed the translation. Third, the two researchers met to resolve any disagreements in the transcript through in‐depth discussion of the data and audiorecording.

### Data Analysis

Transcripts were imported into NVivo 10 (QSR International, Melbourne, Australia) and analyzed using inductive thematic analysis based on steps recommended by Braun and Clarke (2006)—repeated rereading of the data set, generating preliminary codes, searching for and developing candidate themes, and examining and organizing themes. Transcripts were manually coded in a systematic fashion, with initial codes collated to form overarching themes (Braun & Clarke, 2006). Coded text segments for each candidate theme were examined to ensure themes were coherent and accurately reflected the intended meanings evident across the data set (Braun & Clarke, 2006). Preliminary codes and themes were proposed by the primary researcher (VW). Given the subjective nature of qualitative analysis, a reflexive record was kept by the researcher (VW) throughout data collection and analysis to facilitate recognition of assumptions or biases and avert premature interpretations of the data (Mason, 2002). Memos were also recorded regarding the researcher's (VW) reflections and thoughts about emerging themes and relationships between themes (Birks, Chapman, & Francis, 2008). Authors VW and HC independently reviewed all transcripts, with codes and candidate themes examined for agreement, coherence, and accuracy. Any disagreements were resolved following a reexamination of the data. The credibility and trustworthiness of the findings was also established by peer debriefing (Morrow, 2005). Peer debriefing took place with feedback sought from the data collectors who conducted the interviews to ensure codes and themes reflected the sociocultural context of participants. Furthermore, feedback regarding interpretation of the data was regularly solicited from authors SH and IB who have experience with child psychopathology research and qualitative methods.

## Results

Participant characteristics are presented in Table 1, with index trauma characteristics in Supplementary Table 1. Index events included physical and sexual assaults, abductions, witnessing assault, homicide or unexpected death, and road traffic accidents. The majority of caregivers were mothers with low income and employment levels. Time elapsed since the index trauma ranged from 2 to 24 months, with most children having experienced several traumatic events in the past two years. Caregiver interviews lasted 1 hour and 45 minutes on average.

### Qualitative Findings

Four key themes were identified as being of central importance in caregivers’ experiences and efforts to support their child posttrauma (see Supplementary Figure 1 for a thematic map, and Supplementary Table 2 for a detailed presentation of all themes and subthemes). Excerpts are used to illustrate the findings with pseudonyms assigned to all caregivers and children to ensure confidentiality.

#### Theme 1: Caregivers perceive negative impacts of the trauma, primarily due to behavioral indicators in the child

This theme consisted of 5 subthemes (see Supplementary Table 2). Caregivers identified posttrauma changes in their children, and these could be profound.

After the accident he is not right at all … his mind is not stable. … When you say something to him it would stay in his mind … everything of his was stable. But now nothing he does is stable, even if you send him to the shop he will not know why you sent him. (Kuhle, mother, 39 years)

Strong behavioral indicators of poor adjustment were described and identified as being a psychological consequence of the trauma. These included forgetfulness, antisocial behavior, loss of control of bodily functions (e.g., urination), and attention problems.

After the incident she changed, she does not listen, she does not want to go to school, she does not come home in time. If you ask her to do something she will throw tantrums and cry or not do what you told her to do. If she is not going to school she will … go out and only come back about ten p.m. (Bongani, mother, 32 years)

Trauma‐specific responses were also described, including withdrawal, nightmares, and fear of certain places, activities or people.

I have noticed that the children get frightened when there is a knock at the door and they will be the first ones to ask who is at the door, faster than me and their father. (Sisipho, mother, 36 years)

Avoidance mechanisms and safety behaviors in response to the trauma were identified, including avoiding certain areas or activities, refusing to travel alone, and carrying weapons. Caregivers frequently sought others’ impressions of their children's behavior, such as those of teachers, and this provided external validation of concerns. Notably, caregivers primarily relied upon behavioral cues, rather than discussion, to determine their child's emotional response to the trauma. Caregivers often speculated at the causes for their children's behaviors and distress without directly engaging in discussion with their child.

Interviewer: Did you ever talk to Buli about the incident after it happened?

Mother: I never talked to her as I saw that she only gets scared when she sees a male person. (Sanele, mother, 43 years)

#### Theme 2: Varied caregiver support strategies are present, focused on child physical safety as well as emotional coping

Caregivers were sensitive to their children's distress. However, the ways in which caregivers described providing support for children posttrauma were often reactive, and coherent strategies were rarely described. Sometimes supportive behaviors conflicted with each other or were undermined by contextual barriers to support. Predominant emotions expressed by the caregivers were helplessness and defeat, with several caregivers reportedly feeling anxious and unable to adequately care for or protect their children posttrauma. Contributing to these feelings was a lack of support from others and the children's rejection of caregivers’ proffered support.

No one supported me … when you are in trouble [your family] don't want to care for each other, they think you are going to depend on them so they tell themselves that you are going to depend to them while they also have their families. (Bongani, mother, 32 years)

Several subthemes were also identified.

#### Provision of caregiver warmth and responsiveness

This subtheme consisted of eight kinds of caregiver response. Caregivers reported many positive responses intended to alleviate child distress, including encouraging their child to feel safe, normalizing the trauma, and reassuring children that the traumatic event was unlikely to reoccur, although it is notable that caregivers simultaneously emphasized danger and encouraged their child to be vigilant.

I support him by talking to him. I tell him that these things happen, what is important is that he is safe … he needs to move forward and to feel that nothing like that is going to happen again, it will never happen to him all the time even though things happen. (Sinethemba, mother, 37 years)

Support often incorporated religious beliefs and children were encouraged to attend church services, pray, and employ other self‐directed faith‐based coping strategies. Caregivers also promoted a positive perspective of the trauma and encouraged children to think of their future:

Aunt: I would tell him we should pray and sometimes he would say so himself. …He would say “Aunt let's pray, Aunt I prayed last night.” … He would say before we sleep we should pray because he is scared.

Interviewer: How did the family support him, can you explain to me?

Aunt: He was told that God will help him from the situation that he is in, even if he might not stand up again but he must not give up hope because he was not born [paralyzed]. That is how we encouraged him, by giving him those words. (Mihlali, aunt, 45 years)

Caregivers arranged for their child to receive faith‐based protective objects (e.g., rope belts) as a physical symbol of protection and remedy for adjustment difficulties.

I want to take her to St. John's so that they can make a rope [belt] for her and make the water for her to wash with maybe she will be right. … I believe in that church if I wash and drink their water and at night when I am afraid I would shower the house with the same water and open the Bible and sleep. (Bongani, mother, 32 years)

Other indirect ways of showing care were commonly reported by caregivers, including modifying their own behavior to interact with their children in a more sensitive, less punitive manner, and communicating with teachers to make them aware of the source of their children's distress. Providing good physical care, such as ensuring their children were clean or well‐dressed, was also a concern, and some caregivers expressed exasperation that this physical care did not alleviate emotional problems.

#### Caregiver promotion of avoidance

This subtheme consisted of three kinds of caregiver response. Caregivers actively promoted behavioral and cognitive avoidance strategies to cope with the trauma. Caregivers reported avoiding discussion of the event to prevent their children from becoming distressed and avoiding talking about the trauma unless the child initiated the conversation. This meant the trauma was rarely discussed. Caregivers also removed their children from contact with trauma reminders because these were thought to contribute to their children's posttrauma difficulties.

Luthando says as he was hiding behind that house he heard his friend cry once, I suppose that was the time the car hit him. …When he came out, he saw that the car has the hit the yard … and his friend was lying on the ground hit by this car and there was many people. I could not listen to him tell the story, I asked him to stop. I became scared. (Nobuntu, mother, 29 years)

Thinking about the event was thought to signify poor adjustment and caregivers actively encouraged their children to forget the trauma. Consistent with this, caregivers also held expectations that psychological services would make their child forget about the event in order to recover.

Mother: When you are in such a situation you can go to clinics and be sent to counselors and receive counseling and make you forget what had happened. (Olwethu, mother, 46 years)

#### Issuing warnings and caregiver efforts to protect the child from future harm

This subtheme consisted of three kinds of caregiver response. Caregivers reported significant concern for their children's safety following the trauma, and described their community as dangerous and unpredictable. Commensurate with this need to ensure physical safety, caregivers encouraged their children to view the community as dangerous and considered hypervigilant behaviors as a desirable, adaptive posttrauma response. Some caregivers implemented marked changes to their family's daily routines, or their children's school or friendships, in an effort to keep their children safe. Caregivers used warnings and threats of trauma recurrence to encourage adherence to modified routines.

Mother: What I have learned is that if a child is asking for permission to have a nice time with friends I must not allow them because I will not be there to see what they do. Maybe if I was there they would have never stabbed him.

Interviewer: Now that the incident is over, did he ever try to go meet his friends?

Mother: Yes, he did try. … I told him that if you go to your friends you will be stabbed again and this time they will kill you. (Mncedisi, mother, 43 years)

#### Theme 3: Barriers exist to accessing psychological interventions and caregiver involvement is limited

This theme consisted of 5 subthemes. Caregivers often reported supporting their children by seeking medical treatment for their posttrauma difficulties, reflective of the caregivers’ focus on physical or behavioral indicators of change. Caregivers faced considerable challenges in accessing both medical and psychological care for their children posttrauma. The majority of caregivers wanted psychological treatment for their children. However, treatment was often inaccessible because such support was either not available in their area, was too expensive, or included follow‐up appointments that were poorly arranged. Caregivers were often unaware of available treatment, or were not offered a referral following the trauma.

Mother: I only take my child to St. John's to phalaza [traditional medicine] and pray. [If] I have money I can take my child to a specialist doctor so that they can look at her mind. …I can only take her to the doctors that don't cost money.

Interviewer: Generally speaking, what do other families that have children who were in similar situations as Nomsa, do they talk about those things?

Mother: They say they went this way they were not helped, and [then] that way they were not helped, [so] they decided to just sit and let everything go. I tell them that I have not given up on Nomsa. I am still trying. I will stop trying when I get to the place that I am told to go. (Zola, mother, 50 years)

Caregivers perceived counseling to be helpful in remedying their children's adjustment difficulties and an opportunity for the child to discuss the trauma or receive coping advice. Notably, this discussion was thought to be best conducted with a therapist without caregiver participation. Caregivers were often uninvolved in their children's treatment and were unaware of the number of sessions or treatment the child had received. This was reportedly due to limited therapist–caregiver collaboration as well as the caregiver's reluctance to question the child, which could provoke distress.

Mother: [The social workers] visited him in school but I don't know what they asked him and I don't know [the] number of sessions he got, but he would say he was visited by social workers.

Interviewer: Did he tell you about what he had talked about with social workers, how he felt?

Mother: No. He does not like to be asked … so we thought since this was tragic we should not ask him … maybe it pains him when talking about it. (Nobuntu, mother, 29 years)

#### Theme 4: Caregivers’ distress and coping can impact their responses to the children

This theme consisted of eight subthemes. Caregivers reported experiencing significant distress posttrauma. At times this impacted the care and support they were able to provide to their child posttrauma, with some caregivers reportedly feeling too upset to interact with their children or hear details of the event.

I was not all right because I couldn't even look at Cikiziwa … they tested her [for HIV at hospital] and they found out that she was fine. Me too, I became fine and I was able to look at her cause she was saved from what I was very scared of. (Inam, mother, 34 years)

Caregivers reported feelings of blame towards themselves or others for the traumatic event. To manage these feelings, caregivers often sought justice for their children, either through the judicial system or community vigilantism, and believed that this would reduce their own distress or prevent their children from blaming them. At the same time, caregivers also experienced positive psychological changes following the trauma, such as growth in their religious beliefs, readjustment of their life priorities, and a greater appreciation of their child, and gratitude that their children's injuries were not worse.

Caregivers reported receiving social support from friends, neighbors, relatives, members of their church, and colleagues. Some caregivers felt it was important that they talked about the event with others as it deepened their understanding of the event, reducing stress and anxiety.

I decided to talk about it at church so that I get [rid] of it. … [I] told myself it is not my decision to make about what happened, I must get rid of it and go on with life. For example, I did not hide it, if someone asked me what happened I would tell them, that is what helped me most of the time. … I feel much better now. (Babalwa, mother, 29 years)

At the same time, caregivers described using avoidance‐based strategies in an attempt to regulate unwanted thoughts or emotions. Caregivers tried to avoid thoughts about the trauma by keeping busy or actively trying not to think about the event.

I would ask myself what I have done to deserve [this], even if God was punishing me why would he punish me like this? I would ask myself a lot of things. … I end up letting go of it, trying not to think about it because it might kill me. And people always advising me to let go of it for the sake of my children. (Inam, mother, 34 years)

Frequently, caregivers were aware of other children in their community who had experienced similar traumatic events. However, caregivers often reported being unaware of how other families coped. Not only does this reflect the prolific nature of child trauma exposure in the community, it demonstrates that caregivers were often isolated in their distress because support and coping advice from other caregivers whose child had been exposed to a similar trauma was not sought.

## Discussion

Qualitative analysis of caregivers’ accounts of caring for their children posttrauma identified themes relating to caregivers’ perceptions of their children's coping, strategies used to support their children, the impact of the event on the caregiver, experiences of support, and coping strategies employed. The results detail the major challenges faced by caregivers in supporting their children following trauma exposure in a high‐adversity context. Children experienced significant distress posttrauma, and caregivers attempted to support their children with strategies that were, at times, not entirely coherent. Given the numerous barriers to psychological treatment, caregivers often struggled to access treatment for their children and felt anxious and unable to adequately care for or protect their children posttrauma. Caregivers’ involvement in and understanding of their children's psychological treatment was limited. Finally, caregivers’ own distress could be a barrier to providing support. These findings may have implications for family‐based interventions posttrauma.

A major theme emerging from the data related to the caregivers’ understanding of children's psychological responses to the trauma. Caregivers often reported that their children had experienced profound changes following trauma exposure and largely relied on the children's behavioral cues, rather than discussion, to inform their understanding of child coping. This monitoring of behavioral indicators is consistent with qualitative research from low‐risk Western contexts, which also found it to be an important component of parental responses (Alisic, Boeije, Jongmans, & Kleber, 2012; Williamson, Creswell, Butler, Christie, & Halligan, 2016). However, in the latter studies, behavior monitoring was accompanied by simultaneous emphasis on the importance of conversations about child distress, which was not a theme identified in the current sample. Such reliance on behavioral cues could limit caregiver insight into the full range of child symptoms, and it is notable in this respect that poor agreement between parent and child reports of child PTSD symptoms has been found in previous studies (Meiser‐Stedman, Smith, Glucksman, Yule, & Dalgleish, 2007). Promoting caregiver–child conversations about distress could potentially improve caregiver responding to child needs and facilitate appropriate treatment‐seeking.

A second major theme encapsulated different elements of support that caregivers provided in response to the children's traumatic experiences. First, caregivers were proactive in providing support in warm, positive ways, such as offering reassurance and ensuring that their children's teachers would be understanding. This is consistent with previous qualitative research in Western contexts where parents report using warmth and responsiveness to support child recovery posttrauma (Alisic et al., 2012; Williamson et al., 2016), and with interviews with children posttrauma which emphasize general expressions of support and physically warm gestures (hugs) as being important (Alisic, Boeije, Jongmans, & Kleber, 2011). Caregivers encouraged children to hold a positive view of the trauma and their future, which may mitigate against beliefs about persistent negative consequences or their own future vulnerability which are strongly linked to PTSD in children (Meiser‐Stedman, Dalgleish, Glucksman, Yule, & Smith, 2009). Caregivers also acquired faith‐based protective objects as a means of providing physical protection to their child, and such proactive caregiver behaviors may address feelings of helplessness (Galili‐Weisstub & Benarroch, 2005). This is likely to be particularly important given the volatile context and scarcity of other resources. Overall, such expressions of warm care are potentially important; generally high levels of perceived support and positive parental responding have both been associated with lower PTSD symptom levels in children (e.g., Trickey, Siddaway, Meiser‐Stedman, Serpell, & Field, 2012).

It was also striking in the current sample that caregivers promoted avoidance strategies to cope with the trauma, and discussions of the event with the child were limited. Theoretically, this may hinder child adjustment posttrauma by impeding the elaboration and processing of the child's trauma memory and preventing the correction of negative appraisals (Ehlers & Clark, 2000). However, the existing evidence base on this point derives primarily from high‐income, lower risk contexts, where caregiver promotion of avoidance as a coping strategy posttrauma has also been observed, but may be used less widely and for different reasons (Williamson et al., 2016). Caregivers’ advocacy of avoidance‐based strategies in high‐risk contexts likely arises out of necessity and provides physical protection. Moreover, previous research in high‐risk, urban contexts has found positive psychological outcomes in youth who used avoidant, rather than active, coping strategies (Gonzales, Tein, Sandler, & Friedman, 2001; Grant et al., 2000), and it has been suggested that avoidant coping may be less deleterious in environments of chronic or uncontrollable stressors (Gonzales et al., 2001). However, there is extremely limited evidence on this point deriving from high‐risk, LMIC samples. Given the chronic child trauma exposure in some LMIC contexts, this is a significant oversight which should be addressed.

Caregiver support is also strongly related to concerns for child safety. Caregivers in this study tried to protect their children from future harm by emphasizing risks and implementing major socioenvironmental changes. Such responses could be considered as overprotective and potentially maladaptive in a low‐risk context, where they may maintain or enhance child perceptions of threat (Wood, 2006). However, again, systematic evidence evaluating the psychological impact of such parental behaviors in an environment where concerns likely reflect realistic appraisals of future harm is lacking (Eagle & Kaminer, 2014).

Another key theme emerged in relation to caregivers themselves experiencing high levels of distress and helplessness following their child's trauma exposure, which impacted caregiver responses at times, with some caregivers too upset to interact with their children posttrauma. Again, this is consistent with existing evidence that parents perceive their own distress as a potential impediment to supporting their children (Alisic et al., 2012; Williamson et al., 2016), and that parental PTSD is predictive of child PTSD (Morris, Gabert‐Quillen, & Delahanty, 2012). Parents who receive psychological treatment may be better able to cope with the traumatic event and, in turn, provide the necessary support for their child (Hamblen & Barnett, 2003). The assessment of caregiver symptoms during child PTSD treatment, with efforts made to increase the formal support offered to caregivers following child trauma exposure, may be advantageous.

Caregivers in our sample reported little involvement in any psychological interventions received by their child. Family engagement in child treatment posttrauma is recommended because it may result in a reduction of child symptoms and lower dropout rates (e.g., Saxe, Ellis, Fogler, & Navalta, 2012). Engagement in child treatment may further caregivers’ understanding of their children's traumatic experience through co‐construction of the trauma narrative and improve child outcomes by helping children practice their therapeutic coping strategies at home (Cobham, McDermott, Haslam, & Sanders, 2016). The low levels of caregiver engagement in child treatment in the present study may represent an obstacle to child recovery. Previous research has found poor parental engagement in child trauma–focused cognitive behavioral therapy (CBT) in Zambia, leading to recommendations that parents be offered an additional session to provide psychoeducation and improve parental involvement (Murray et al., 2014). Additional research is needed to investigate effective means of improving caregiver engagement in child psychological treatment in contexts where such involvement is limited.

The current study provided perspectives on an important, yet understudied, group, in examining responses to child trauma in a context of extremely high risk. We recruited a sample of caregivers of children with diverse experiences of trauma, and took steps to ensure the quality of local data collection and analysis through a robust system of translation, training, and supervision. Nonetheless, there are limitations that must be considered. First, this study only included female caregivers, consistent with the cultural context of South Africa where fathers are often absent and much of the burden of care is placed on mothers or other female relatives (Budlender & Lund, 2011). Future examination of the experiences of male caregivers could provide valuable insight. Second, only caregiver‐report measures were collected and future research should also include child informants. Third, although efforts were made to ensure data analysis was reflective of the sociocultural context, it was not possible to conduct respondent validation. Finally, although steps were taken to ensure data integrity, bias may have been introduced because the interviews were conducted in Xhosa and translated to English.

Overall, our findings are striking both in the similarity in caregiver responding in our study to that found in studies of low‐risk Western samples, and in the differences in emphasis that manifest in the current sample. Thus, caregiver promotion of avoidance, limitations on trauma discussions with children, desire to provide protection by emphasizing risk, and impediments created by caregiver distress have each been identified in previous studies but were particularly strongly expressed in the current, high‐risk sample. Equally, provision of caregiver warm responsiveness appears to be broadly typical of child trauma samples, but may be expressed in distinctive ways (e.g., the emphasis on religious symbolism in the current study). Future systematic investigation of potential contextual variation in caregiver support is needed, including in relation to possible impacts on child adjustment, and may yield specific targets for dyadic interventions. The current findings also illustrate the considerable challenges faced by caregivers in high‐risk contexts where resources are scarce, and the barriers that exist to accessing treatment. This suggests a pressing need for accessible psychological interventions in LMIC, and for efforts to improve caregiver engagement in child treatment which may help to improve child adjustment and overall family functioning posttrauma.

## Supporting information

Figure S1. Thematic map of themes and subthemes following thematic analysis. Themes are displayed in boxes, with subthemes underlined. Lines represent the associations between themes/subthemes.

Table S1. Participant trauma characteristics

Table S2 Themes and sub‐themes following thematic analysis
