# Supplementary material for: Caregiver Responses to Child Posttraumatic Distress: A Qualitative Study in a High‐Risk Context in South Africa
Source: J Trauma Stress. 2017 Oct 27;30(5):482–90. doi: 10.1002/jts.22215 (PMC5698750; doi:10.1002/jts.22215)
Supplement: Supplementary file 1 — Figure S1. Thematic map of themes and subthemes following thematic analysis. Themes are displayed in boxes, with subthemes underlined. Lines represent the associations between themes/subthemes. [file JTS-30-482-s001.docx]

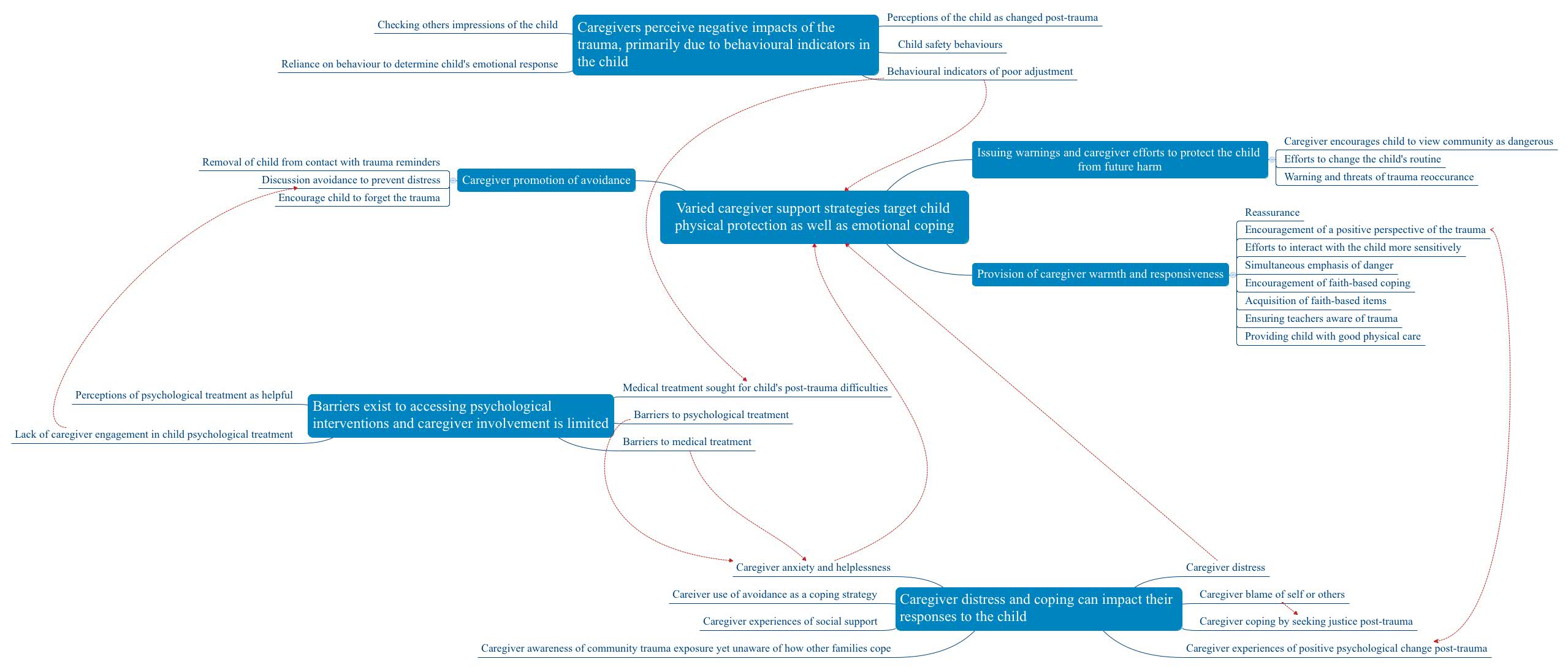


*Supplementary Figure 1:* Thematic map of themes and subthemes following thematic analysis. Themes are displayed in boxes, with subthemes underlined. Lines represent the associations between themes/subthemes.
