# Supplementary material for: Caregiver Responses to Child Posttraumatic Distress: A Qualitative Study in a High‐Risk Context in South Africa
Source: J Trauma Stress. 2017 Oct 27;30(5):482–90. doi: 10.1002/jts.22215 (PMC5698750; doi:10.1002/jts.22215)
Supplement: Supplementary file 2 — Table S1. Participant trauma characteristics [file JTS-30-482-s002.docx]

Supplementary Table 1

*Participant trauma characteristics*

| Caregiver pseudonym | Caregiver interviewed | Child age | Child sex | Trauma experienced |
| --- | --- | --- | --- | --- |
| Anele | Mother | 15 | Female | Witnessed a death |
| Fezeka | Mother | 16 | Male | Physical assault |
| Inam | Mother | 9 | Female | Sexual assault |
| Lulama | Grandmother | 11 | Female | Physical assault |
| Mandisa | Mother | 16 | Female | Witnessed a death |
| Nikelwa | Mother | 15 | Female | Physical assault |
| Nobuntu | Mother | 10 | Male | Witnessed a death |
| Mncedisi | Mother | 15 | Male | Physical assault |
| Sisipho | Mother | 9 | Male | Armed robbery |
| Olwethu | Mother | 11 | Male | Sexual assault |
| Sanele | Mother | 6 | Female | Witnessed physical assault |
| Vela | Mother | 15 | Male | RTA |
| Zola | Mother | 11 | Female | Abducted |
| Sinethemba | Mother | 11 | Male | Abducted |
| Thembeka | Aunt | 8 | Female | RTA |
| Mihlali | Aunt | 9 | Male | Physical assault |
| Babalwa | Mother | 14 | Female | Sexual assault |
| Bongani | Mother | 9 | Female | Sexual assault |
| Gcobisa | Mother | 11 | Female | RTA |
| Kuhle | Mother | 9 | Male | RTA |

*Note*: RTA = Road traffic accident. Trauma experienced = primary traumatic event discussed by the caregiver in the

qualitative interview.
