# Supplementary material for: Caregiver Responses to Child Posttraumatic Distress: A Qualitative Study in a High‐Risk Context in South Africa
Source: J Trauma Stress. 2017 Oct 27;30(5):482–90. doi: 10.1002/jts.22215 (PMC5698750; doi:10.1002/jts.22215)
Supplement: Supplementary file 3 — Table S2 Themes and sub‐themes following thematic analysis [file JTS-30-482-s003.docx]

Supplementary Table 2

*Themes and sub-themes following thematic analysis*

| *Theme and sub-theme and number of participants disclosing the sub-theme* | |
| --- | --- |
| **Theme 1: Caregivers perceive negative impacts of the trauma, primarily due to behavioural indicators in the child.** |  |
| *Perception of the child as changed post-trauma (12)* | Onele grew up a quiet child, she was alright until the accident, then her mind totally changed. I do not get her at all, she does not know if she is at school or not, she does not write at school. (Lulama, grandmother, 57 years) |
| *Behavioural indicators of poor adjustment (10)* | Interviewer: Did you notice any change in him after the incident? Mother: He did not play with his friends, even when they came to him, he would not look interested… He lost [his] appetite, he would not eat his breakfast and would not eat his lunch, he would come back with his lunchbox the same way it was when he left. He lost weight and had bad skin. (Nobuntu, mother, 29 years) |
| *Reliance on behaviour to determine child's emotional response (11)* | You would see that she does not feel like she is a child that belongs here, you would see that she is hurt...she would be as if she is not from here. Even if someone would enter the house, they would think that she is not from here. (Bongani, mother, 32 years) |
| *Child safety behaviours (4)* | Phila did not feel better…. he [now] likes carrying a knife and when I asked him what he is doing carrying a knife all the time, he said he is preparing for those shits if they come to [him] unexpectedly, he would stab them. (Fezeka, mother, 31 years) |
| *Checking others impressions of the child (8)* | Everything of his was stable but now nothing he does is stable.... Even his class teacher…told me that Bhutana is not the same. You tell him this, he will say something else… and when you tell him something he will not remember. (Kuhle, mother, 39 years) |
|  |  |
| **Theme 2: Varied caregiver support strategies target child physical protection as well as emotional coping.** |  |
| **Provision of caregiver warmth and responsiveness** |  |
| *Reassurance (13)* | Mother: I told her that another person will not be like the person she saw stabbing her aunt. I tell her that people are different, not all of them are dangerous. (Sanele, mother, 43 years) |
| *Encouragement of a positive perspective of the trauma (14)* | He must not think his life is [in] that wheelchair. He must know that life goes on, it does not end [now that he uses] the wheelchair. More especially, that he has to still go to school and it is something that we are working on. (Mihlali, aunt, 45 years) |
| *Efforts to interact with child more sensitively (15)* | I knew she will not be right, so I decided to be soft and be next to her all the time. I did not force her to go to school and I did not leave her to go to work. I wanted to be close to her and spend time with her. I stayed with her and I saw that she is becoming right. (Babalwa, mother, 29 years) |
| *Simultaneous emphasis of danger (3)* | I told her that person is not here as he was sentenced to life in prison and she said that she does not really trust that, maybe that person can escape and kill her. I told her that he is not here now… but I told her that if she ever sees him, she must run. (Zola, mother, 50 years) |
| *Encouragement of faith-based coping (13)* | I make sure that I take him to the nearby church and make sure he is in church...I wish he would go to church and learn church things… There is also Sunday school there so he [can] learn to pray, so that when something bad happens to him, he [can] pray for himself. (Nobuntu, mother, 29 years) |
| *Acquisition of faith-based items (6)* | Mother: My child has no problem. I also have no problem because I have faith in what I am using. I just encourage them that they should drink the water from the church three times a day and they must also use the soap. Since I have faith in that, I can see they do not have a problem.  Interviewer: The water and the soap are protecting your family?  Mother: Yes. (Mandisa, mother, 43 years) |
| *Ensuring teachers aware of trauma (12)* | Mother: I [told] their father that we must follow them to school to look how they are doing and when we got there the teachers said they didn’t notice anything…I was worried that maybe the teacher will be busy teaching and they are not concentrating [because they’re] thinking about what happened in their home. Maybe the teacher gave him some paper and he does nothing on it, I did not want the teacher to have difficulties with them and I did not report what happened. (Sisipho, mother, 36 years) |
| *Providing child with good physical care (14)* | Onele is not writing at school...every time I go to school, [I'm told] this child does not write. She does nothing and I am the one who does everything for her. I made means for her to have a birth certificate, Government grant money, I even bought her school things. She is becoming worse as she grows up… I buy tracksuits, I do her hair, but the problem is she does not write. The teachers say she is clean, there is nothing that shows she is not taken care of. (Lulama, grandmother, 57 years) |
| **Caregiver promotion of avoidance** |  |
| *Discussion avoidance to prevent distress (9)* | When I talk to her, especially about the incident that nearly happened to her, she cries and I decide to let it go, seeing that she is hurting, I would let it go. (Bongani, mother, 32 years) |
| *Removal of child from contact with trauma reminders (7)* | On our way on the veld to Philippi, there were [some] bushes with stones and he said "look Mom, this is where my uncle and dad [died]," I tried to disturb him and said let’s go. (Kuhle, mother, 39 years) |
| *Encourage child to forget the trauma (10)* | Mother: I support him...I tell him not to nurse the problem he was in. He must let things go, because if he does not then other important things in his life will come in a stand still. Interviewer: Things like what? Mother: For example, he is a school child, maybe he would be thinking about the incident while doing his school work and the thought would disturb him. (Vela, mother, 43 years) |
| **Issuing warnings and caregiver efforts to protect the child from future harm** |  |
| *Caregiver encourages child to view community as dangerous (17)* | I told her...people in Cape Town are shot like birds. You enter your home and it might happen that there is someone hiding wanting to shoot someone and they will shoot you because they are angry [that] they did not get the person they want…I tell them that the doors must always be closed because [our neighbourhood] is not cool and they listen to me. (Mandisa, mother, 43 years) |
| *Warnings and threats of trauma reoccurrence (14)* | I tell her that if you keep on doing what you doing wandering at night you will get raped, have your womb removed before time, or even have HIV and not a normal life. (Thembeka, aunt, 38 years) |
| *Efforts to change the child's routine (16)* | I was thinking he would change because at the hospital I told him to let go of his friends because he nearly died because of his friends but he did not listen to me. The day he arrived [home] from the hospital he went to his friends. (Fezeka, mother, 31 years) |
| **Theme 3: Barriers exist to accessing psychological interventions and caregiver involvement is limited.** |  |
| *Medical treatment sought for child's post-trauma difficulties (9)* | When she got home she was terrified and said “Mom I saw this and that” ...she was not right that day, she said she had a picture of what she saw [in her mind] ...and I took her to the clinic and I told them about the incident, they said that they will give her [paracetamol] and pills for her to be right so that she can be able to sleep. She got the pills and drank them, she went to school and she was alright. (Mandisa, mother, 43 years) |
| *Barriers to medical treatment (16)* | We went home to fetch Lunga and took him to the hospital.... While in hospital we were not received well ... there were other people there who were also injured, others were laying on the floor bleeding but [they] were not attended to. I also saw that time went by without us being attended to and my son is in pain. (Mncedisi, mother, 43 years) |
| *Barriers to psychological treatment (17)* | The child was taken to counselling. She was given dolls there to show what happened and she did, she showed them and…. they said they were going to call us, but they never did...she never went again. [The counsellors] went to school to collect reports about her, they wanted to know how she was after the incident. They never came back again. (Babalwa, mother, 29 years) |
| *Perceptions of psychological treatment as helpful (10)* | What I have always wished for, but I do not know how to go about it, is that my child meets someone like a social worker who tell him that in order to be right you need to stop smoking and drinking and things like that... so he can be able to save himself from those things like [being stabbed again]. (Mncedisi, mother, 43 years) |
| *Lack of caregiver engagement in child psychological treatment (7)* | Mother: I did not attend [his counselling]. I don't even know what he said there. I really want the social workers because I don't like the child who is not open, he could be in trouble...  Interviewer: So you wish that the sessions include you? Mother: Yes.  Interviewer: Because the ones from school, you are not sure how it went?  Mother: Yes, I am not sure, truly. (Nobuntu, mother, 29 years) |
| **Theme 4: Caregiver distress and coping can impact their responses to the child.** |  |
| *Caregiver distress (10)* | Interviewer: As for now what do you think about the incident, is there something you think of it?  Mother: It has not faded because even when I am asleep I dream about it happening to her...it does not go away. (Bongani, mother, 32 years) |
| *Caregiver anxiety and helplessness (16)* | Mother: I do think they might come back, as they know that they have taken the gun from us so there is nothing we can use to protect ourselves. They can come and break in again. (Sisipho, mother, 36 years) |
| *Caregiver use of avoidance as a coping strategy (9)* | The reason why I sell things is that I want people to come by to give me that chance to interact with people, it makes me forget…I like selling thing that the children like to buy, like sweets. I know every minute they would come buy 10c sweets. I even make chicken feet, that keeps me busy a lot, it makes me forget for a while and I don’t get depressed. |
| *Caregiver experiences of positive psychological change post-trauma (13)* | Interviewer: How do you feel about your belief now? Mother: I still believe….[My faith] has grown because there are many things, many challenges and tests. When they happen I cannot just say, no God does not love me anymore… indeed God has helped in that He just wants me to see His grace. (Nobuntu, mother, 29 years) |
| *Caregiver blame of self or others (16)* | Interviewer: What is making you angry? Mother: What he did to my child. Letting someone in my house, not knowing he had other plans. You regard him as a child [and] all along he is a criminal. I blame myself for this, I will stop blaming myself when he is behind bars so that my child can be at peace. Interviewer: Why do you blame yourself?  Mother Because his mother called saying her child wanted to visit and I said yes. I should have never said yes...It should have never happened. (Olwethu, mother, 46 years) |
| *Caregiver coping by seeking justice post-trauma (11)* | Interviewer: You mentioned that you will be at peace only when the boy is arrested. Mother: Yes, only when he is arrested….Because that boy came to me, so I feel my son will feel that my mom brought someone home and [he] abused me and she did nothing about it. That is why I feel he should be arrested. At least when my child hears that his mother did something it he will feel better. (Olwethu, mother, 46 years) |
| *Caregiver experiences of social support (15)* | I went to church and at home we would talk about it... I felt like there are people next to me. Even the women in the community would come and tell me when the trial starts I should tell them they will come with me to court for moral support. (Babalwa, mother, 29 years) |
| *Caregiver awareness of community trauma exposure yet unaware of how other families cope (11)* | Interviewer: OK, now generally asking, what do other families in your community do to overcome incidents that happen to their children, incidents similar to that of Esihle? Mother: I really don’t know, I’ve never seen any family deal with their problems, and there is no one who ever told me their problems…there is no one that comes to you even if they heard what had happen to you to advise you to do this or that way...I never got that. (Babalwa, mother, 29 years) |
|  |  |
